# Effects of xenobiotics on total antioxidant capacity

**DOI:** 10.2478/v10102-012-0019-0

**Published:** 2012-09

**Authors:** Carlos Kusano Bucalen Ferrari

**Affiliations:** Biomedical Research Group, Instituto de Ciências Biológicas e da Saúde (ICBS), Campus Universitário do Araguaia, Universidade Federal de Mato Grosso (UFMT), Av. Gov. Jaime Campos, 6390, Distrito Industrial, Barra do Garças, 78.600-000, MT, Brazil

**Keywords:** total antioxidant capacity, free radicals, GSH, trolox, xenobiotics, smoking, alcohol

## Abstract

The objective of this article was to review the effects of xenobiotics on total antioxidant capacity (TAC). Measurement of TAC is appropriate for evaluation of the total antioxidant defenses of blood, cells, and different kinds of tissues and organs. TAC is reduced by alcoholism, smoking, and exposure to radiation, herbicides, carbon monoxide, carbon tetrachloride, lead, arsenic, mercury, cadmium, aluminum, and other toxic elements. The test is also an important tool in evaluating environmental and occupational exposure.

## Introduction

The involvement of unstable free radicals and reactive species from oxygen (ROS), nitrogen (RNS), and chlorine in cell and tissue damage is well established (Vladimirov & Proskurnina, 2010; Ferrari, 2000).

A free radical (FR) is any molecule that has one or more incomplete orbitals. A FR can gain electrons, oxidizing another atom/molecule or lose them, thus reducing an element. Some reactive oxygen species (ROS) are FR [superoxide anion (O_2_^•–^), hydroxyl radical (^•^OH), nitric oxide (NO^•^), peroxynitrite (ONOO^–^), though others are not [hydrogen peroxide (H_2_O_2_), singlet oxygen (^1^O_2_)] (Halliwell, 2011).

FR, ROS and RNS have been associated with more than a hundred diseases or pathophysiological events since they cause changes of lipids (lipid peroxidation), proteins (protein peroxidation), nucleic acids (DNA or RNA oxidation), and carbohydrates (glycosilation) (Vladimirov & Proskurnina 2010; Ferrari *et al.*, 2009).

Since the 1960's, many research groups have been evaluating oxidative and nitrosative stress and the antioxidant defenses by measuring the activity of antioxidant enzymes such as superoxide dismutase (SOD), catalase (CAT), glutathione redutase (GSH) and glutathione peroxidase (GPx). As a consequence of oxidative phosphorylation, 3–5% of oxygen in converted to the free radical superoxide anion (O_2_^•–^) by mitochondria. As a further step, SOD and GPx convert O_2_^•–^ into hydrogen peroxide (H_2_O_2_). However, as H_2_O_2_ is also a toxic reactive oxygen species, it should also be modified to inocuous water and atomic oxygen by the enzyme CAT.

Since 1993 many interesting tests have been proposed in order to measure the total antioxidant capacity (TAC) of a biological sample (blood, saliva, urine, feces), food or vegetable extract or of living tissues and organs (Cao *et al.*, 1993; Miller *et al.*, 1993; Benzie & Strain, 1996; Ferrari, 2008). Yet these TAC tests measure only hydrophilic antioxidants and they are strongly influenced by the uric acid in the sample.

Although the evaluation of tissue/organ total antioxidant capacity *per se* is not sufficient to assess the level of oxidative/nitrosative stress, it is valuable since it provides a gross estimation of how the body can react against oxidative and nitrosative injuries (Filho *et al.*, 2007; Constantini & Verhulst, 2009).

The aim of this article was to review the health effects of several xenobiotics and environmental hazards on TAC of living organisms, especially mammals and man.

## Total antioxidant capacity and pharmaceuticals

Xenobiotic is any factor, substance or element, exogenous to the organism (alcohol, drugs, environmental and occupational contaminants, pesticides, smoking, heavy metals). Many xenobiotics can be toxic to cells, tissues and organs, constituting environemntal and occupational health problems.

In order to know if a therapeutic drug can preserve or consume the antioxidants of a patient, TAC determination is done in medicinal drugs. Thus the perioperative anesthetics dopamine (1080±162 mmolET/L), propofol (100±18 mmolET/L), dobutamine (80±16 mmol ET/L), and noradrenaline (62±16 mmolET/L) were found to present higher TAC values (Mantle *et al.*, 2000). However, the antioxidant response to occupational exposure to anesthetic gases is different. Human exposure to these gases increased mononuclear leukocyte DNA damage and oxidative stress and reduced TAC of operating room workers (Baysal *et al.*, 2009). Measured by TAC, captopril used as antihypertensive drug has also antioxidant activitiy (Benzie & Tomlinson, 1998), and so have fluvastatin and simvastatin, commonly used in treatment of hypercholesterolemic patients (Franzoni *et al.*, 2003). Another statin, atorvastatin, has also been found to enhance plasmatic TAC and decrease oxidative stress in coronary artery disease dislipidemic patients (Buyukhatipoglu *et al.*, 2010). Natural flavonoids like pomiferin also protected rats against ischemic-reperfusion kidney injury by decreasing lipid peroxidation and GPx, and improving the SOD and TAC levels (Bartošíková *et al.*, 2010).

Paracetamol administration induced liver damage associated with increased lipid peroxidation, decreased catalase, GSH, and total thiols (Ashok-Kumar *et al.*, 2010). Oral intake of paracetamol over 14 days was associated with a drastic reduction of blood TAC in healthy subjects (Nuttal *et al.*, 2003). In a similar manner, administration of the cycloxygenase inhibitor indometacin induced depletion of GSH, stomach epitelhium mucin, and total antioxidant capacity, concomitantly increasing lipid and protein peroxidation in gastric mucosa of mice (Adhikary *et al.*, 2011).

## Effects of chemical poisoning on TAC

Carbon tetrachloride (CCl_4_), one of the most potent liver toxins, presents a toxicological dose-response effect characterized by oxidative stress and lipid peroxidation events that induce reduction of liver TAC, liver degeneration, necrosis and fibrosis (Dianzani & Ugazio, 1973; Ugazio *et al.*, 1973; Hassan *et al.*, 2003; Mahmoud & Hijazi 2007; Wu *et al.*, 2008).

Many heavy metals and other inorganic elements can contaminate the environment, causing bioaccumulation in plants and animals, intoxication in birds and other animals, including humans. The most important pathological mechanism is metal-induced oxidative stress which affects bivalves molluscs, aquatic insects, fishes, birds, and humans (Taylor, 2009; Xie *et al.*, 2009; Zheng *et al.*, 2011; Koivula & Eeva, 2010; Verlekar & Chainy, 2008; Guilherme *et al.*, 2008; Kobal *et al.*, 2008). The toxicity effects of contaminating elements or compounds are presented in Table 1.

**Table 1:** Effects of chemical elements or compounds on total antioxidant capacity.

Element or compound	Effect	Reference
Aluminum	Lipid peroxidation, ↑CAT, ↓GPx Lipid peroxidation, ↓CAT, ↓SOD ↓GPx in brain Lipid peroxidation, ↓CAT, ↓GST, ↓GSH and induced degeneration and death of testis and sperm	Özkaya *et al*. (2010) Shati *et al*. (2011) Yousef and Salama (2009)
Arsenic	↑Free radicals; ↓GSH; impairment of glucose metabolism; Oxidative stress and ↓TAC	Flora (2009) Wu *et al.* (2001)
Cadmium	Oxidative stress-induced nephrotoxicity and neurotoxicity; ↑Free radicals in liver; ↑ lipid peroxidation and GPx, ↓GSH; inhibited CAT	Flora (2009) Son *et al*. (2009) Eybl and Kotyzová (2010)
Carbon tetrachloride (CCl_4_)	↑Free radicals and lipid peroxidation in liver; ↓ TAC in liver; induces liver necrosis and fibrosis; ↑GPx, ↓GSH; inhibited CAT	Hassan *et al.* (2003); Mahmoud and Hijazi (2007); Wu *et al*., (2008)
Cobalt	Oxidative stress-induced heart damage	Flora (2009)
Copper	↓TAC and ↑iNOS causing mitochondrial failure and death of neurons and astrocytes	Reddy *et al.* (2008)
Lead	Preferential accumulation in hippocampus, thalamus, parietal cortex, and striatum, inducing lipid peroxidation Increased expression of SOD and CAT to counteract the oxidative stress and ↑lipid peroxidation in hippocampus and cerebellum N-acetyl-cysteine improve TAC of brain decreasing lipid peroxidation damage by lead ↓Nitric oxide causing hypertension and neuronal mitochondiral impairment ↑Lipid peroxidation and ↓ GST, CAT and SOD in bone of rats	Villeda-Hernández *et al.* (2001) Bennet *et al*. (2007) Nehru and Kanwar (2004) Chen *et al*. (2000) Alghasham *et al*. (2011) Payal *et al*. (2009)
Manganese	↑Oxidative stress, ↑LDL oxidation, ↑DNA oxidation and ↑TAC	Komatsu *et al*. (2009)
Mercury	Oxidative stress in brain, kidney and liver; liver and kidney damage; ↓SOD, ↓GPx, ↓GSH; ↑Oxidative stress; ↑Lipid peroxidation; ↓sperm motility	Agarwal *et al*. (2010) Rao and Gangadharan (2008)
Nicotine	DNA oxidation and ↓TAC	Toklu *et al*. (2010)
Ozone	↓TAC	Bocci *et al*. (1998)

Lead exposure was associated with hypertension, higher activation of angiotensin-converting enzyme, increased levels of lipid peroxidation, and lower levels of nitric oxide and total antioxidant capacity (Alghasham *et al.*, 2011).

As metallothionines (MTs) are important metal-scavenging proteins protecting cells against cadmium, mercury, zinc and copper cytotoxicity (Kang, 2006; Aschner *et al.*, 2006; Agarwal *et al.*, 2010), newer studies are important to address the potential of MTs to improve cell and tissue TAC in patients.

## Alcohol, oxidative stress and total antioxidant capacity

Moderate alcohol drinking can increase TAC, whereas daily and higher ingestion of alcoholic beverages reduce blood TAC (Brighenti *et al.*, 2005) reinforcing the knowledge that chronic alcohol intake contributes to oxidative stress, lipid peroxidation, mitochondrial damage and failure, depletion of GSH cytosolic stores, liver injury and hepatocyte apoptosis (Liu, 2004; Cederbaum *et al.*, 2009). Lung injury due to alcoholism is in part derived from a proinflammatory reaction and an intense oxidative stress linked to depletion of GSH stores (Burnham *et al.*, 2003; Boé *et al.*, 2009). Alcoholic patients presented with lower SOD and GPx activities and higher values of lipid peroxidation yet normal CAT activity (Huang *et al.*, 2009). After alcohol conversion to acetaldehyde, lipid peroxidation reactions are triggered; further, alcohol metabolism in hepatic microsomal systems is also associated with increased production of reactive oxygen species (Pisa *et al.*, 2010). This is in accordance with other studies in which there was no significant change in plasma TAC after alcohol drinking (Bhardwaj *et al.*, 2008).

## Occupational and environmental xenobiotics: effects on TAC

### Smoking and depletion of total antioxidant capacity

Environmental pollutants can also affect TAC. In a study in Lodz, Poland, smokers presented lower plasma TAC values in comparison to non-smokers (Goraca & Skibska, 2005; Goraca & Skibska, 2006). It is very important to note that among smokers urinary TAC was not a biomarker of oxidative/nitrosative stress, but 8-hydroxy-2’-deoxyguanosine, a DNA oxidation product, and the advanced glycation end products from oxidation of carbohydrates were both excellent urinary oxidative stress biomarkers in healthy smokers (Campos *et al.*, 2011). This could be explained by the presence of many antioxidants in urine (e.g. uric acid), which prevent depletion of TAC (Benzie & Strain, 1996; Rice-Evans, 2000). Nicotine administration to rats had many deleterious effects such as DNA oxidative damage, induction of proinflammatory cytokines (TNF-α and IL-1β) and decrease of TAC in urogenital organs (Toklu *et al.*, 2010). Smoking has been also related to increased lipid peroxidation and reduced SOD, CAT, GSH and GST levels in kidneys and liver (Ramesh *et al.*, 2010). In the same study, GPx activity decreased in the liver but increased in the kidney of rats exposed to smoking. Among healthy young adult smokers, two tests of TAC and GSH levels were reduced compared to non-smoking subjects, whereas lipid peroxidation and oxidized LDL cholesterol were found to be increased in smokers compared to controls (Bloomer, 2007). The effect of smoking during pregnancy was studied in plasma, kidneys, brain, liver and lungs of rats. The authors found reduced TAC levels in liver, kidneys and brain of non-pregnant rats, whereas increased TAC was recorded in the lungs of this group. In the same study, in pregnant rats exposed to cigarette smoking, reduced TAC was observed in kidneys and increased TAC values in lungs and brain with no significant change of TAC in liver and plasma (Florek *et al.*, 2009). Periodontitis patients presented higher levels of lipid peroxidation associated with lower levels of TAC and smoking enhanced this deleterious effect (Guentsch *et al.*, 2008). A closely related study also found correlation between reduced levels of TAC and the presence of gingivitis and periodontitis in smokers compared to non-smokers (Al-Bayati *et al.*, 2011). Another interesting study reported that smoking was associated with increased lipid peroxidation levels in mothers and their fetuses and decreased TAC in both groups (Chelchowska *et al.*, 2011). This explains the reduced TAC in patients with chronic obstructive pulmonary disease (COPD), an effect that was not associated with disease severity (Rahman, 2000).

### Occupational hazards and TAC

In a study of a Taipei population group, Taiwan, exposure to arsenic was found to induce oxidative stress along with an inverse association between arsenic blood levels and TAC (Wu *et al.*, 2001). Exposure to ozone (O_3_) can also reduce plasma TAC by 20% (Bocci *et al.*, 1998). Another interesting study showed drastic reduction of plasma TAC among bricklayers exposed to cement and related materials (Pournourmohammadi *et al.*, 2008). Workers exposed to dust from tobacco leaves presented reductions in TAC from 15.5% to 31% (Swami *et al.*, 2006).

Nevertheless, there are some pollutants that do not cause changes in TAC. In carbon monoxide poisoning from incomplete combustion of fossils, despite intense production of carboxyhemoglobin and peroxidative products, the exposed patients did not reveal significant changes in blood TAC (Kavakli *et al.*, 2011). Due to inhibition of the mitochondrial-membrane complex IV system, CO poisoning causes hypoxia, lipid peroxidation, and neurological sequelae (Garrabou *et al.*, 2011). Similar mechanisms are operative in exposure to herbicides. Picloram and triclopyr herbicides diminished cell viability and decreased expression of neuroprotective genes and mitochondrial electron transport genes in neurons (Reddy *et al.*, 2011). Administration of the insecticide triazophos induced lipid peroxidation and glutathione-S-transferase activity (GST) with subsequent depletion of GSH and TAC, resulting in progressive liver degeneration (Jain *et al.*, 2010).

Radiation exposure induced inflammation, increased oxidative stress and decreased TAC values in human lymphocytes (Lee *et al.*, 2010). It has been suggested that TAC levels were inversely correlated with cytogenetic radiation-induced damage in man, which means that antioxidant capacity is a protective factor against radiation hazard effects (Köteles *et al.*, 2001).

It is important to note that depending on the type and intensity of exposure, TAC levels can be enhanced, not reduced, since cells and tissues can react by the hormesis response producing more heat shock proteins (HSPs) and activating the nuclear antioxidant response element which is further responsible for the increase in TAC levels. This could explain why exposure to magnetic fields has been correlated to increased TAC levels in male workers (Sirmatel *et al.*, 2007). Nevertheless, other studies which evaluated the effects of magnetic fields on antioxidant response reported increased oxidative stress, and lipid peroxidation and decreased TAC in rat brains, especially in aged animals (Akdag *et al.*, 2010; Falone *et al.*, 2008). Exposure to low-frequency magnetic-field induced depletion of GSH and TAC associated with increased levels of lipid peroxidation and production of reactive oxygen species in liver and heart, depending on timing and distance from the field (Canseven *et al.*, 2008; Goraca *et al.*, 2010).

Exposure to eletromagnetic radiation of 900MHz from mobile phones induced lipid peroxidation in the hippocampus and brain cortex of rats as well as oxidative stress and histopathological changes in the rats’ endometrium, effects which were reversed by antioxidants (Köylü *et al.*, 2006; Guney *et al.*, 2007). Further studies should address the question whether radiation frequency from cell phones can induce oxidative/nitrosative stress and depletion of TAC in human subjects.

It should be noted that the tecniques for determination of TAC in blood and biological fluids are still very recent. Thus potential clinical correlations between TAC and disease are yet in progress. Similarly, in many pathophysiological conditions the relationship with TAC levels has yet to be established. In a study with different degrees of injury, there was no correlation between plasma TAC levels and severity of lesions in patients with burns (Farriol *et al.*, 2001). In other research reports there was a reduction in plasma TAC levels in patients with burn injuries (Nagane *et al.*, 2003). Borisenkov *et al.*, (2007) revealed that TAC of saliva was increasing during the period from 3 AM to 7 AM, and presented but small changes between 12 AM and 12 PM. This should be considered for the best time to collect samples for TAC testing.

## Conclusions

Alcohol, smoking, heavy metals and toxic elements, some pesticides, some occupational exposures, and eletromagnetic and nuclear radiations can decrease TAC, rendering subjects less resistant to oxidative and nitrosative injuries and subsequent diseases. More research is needed to address the role of antioxidant supplementation in xenobiotic exposure and disease prevention.
